# Brooding Phylogenomics: Target‐Capture Probe Sets for the Analysis of Ultraconserved Elements in the Peracarida

**DOI:** 10.1111/1755-0998.70078

**Published:** 2025-11-10

**Authors:** Andrew G. Cannizzaro, David J. Berg

**Affiliations:** ^1^ Department of Biology Miami University Oxford Ohio USA; ^2^ Department of Biology Miami University Hamilton Ohio USA

**Keywords:** Amphipoda, Crangonyctidae, Hyalellidae, Isopoda, Sphaeromatidae, *Thermosphaeroma*, UCEs

## Abstract

Sequencing via target capture has been used to great effect in phylogenetic studies of organisms such as insects, arachnids and vertebrates. However, other taxa have received limited genomic attention despite their diversity and the intensity of research on such groups. Here, we describe generalised probe sets targeting ultraconserved elements (UCEs) for members of the crustacean orders Amphipoda and Isopoda in the superorder Peracarida. These sets employ ~10,000–100,000 probes targeting up to 10,000 loci. In silico analyses of these probe sets recovered an average of 5087 loci, while an average of 4633 was retained post‐filtering. Phylogenetic analysis of these datasets resulted in well‐supported trees that align with previously reconstructed relationships among the taxa selected while also providing resolution of previously uncertain nodes. Following the in silico analysis, an in vitro analysis targeting several amphipod and isopod families was conducted. This analysis extracted up to 4864 unique loci from the taxa sequenced, with an average of 1897 loci among all taxa. This represents an order‐of‐magnitude increase versus previously published sets, which were only able to recover < 250 UCEs among peracarid taxa. Phylogenetic analyses of the data generated in vitro resulted in well‐supported trees that were resolved at both shallow and deep taxonomic levels. Both analyses demonstrate the utility of these probe sets for phylogenomic research within the Peracarida. Additional attention to members of the superorder using target enrichment will doubtlessly assist in resolving poorly understood aspects of their evolutionary history and expand current knowledge of this group.

## 1 Introduction

With over 20,000 described species, the superorder Peracarida represents one of the most diverse groups of crustaceans; peracarids boast a worldwide distribution, with representatives recorded from virtually all varieties of aquatic systems and even several terrestrial systems (Poore 2005; Väinölä et al. 2007; Wilson 2007; Poore and Bruce 2012; Arfianti and Costello 2020). Their ubiquity can also be explained by the age of the superorder, which likely originated during the Devonian; orders such as Isopoda and Amphipoda split during the Carboniferous and major pulses of diversification occurred during the Jurassic/Cretaceous (Copilaş‐Ciocianu et al. 2020; Robin et al. 2021). As a result, peracarid crustaceans have the potential to serve as excellent model organisms for addressing evolutionary and biogeographic hypotheses across a variety of time scales. Peracarids are present in a broad range of environments, and in many cases, they have occupied these environments for a substantial amount of time, utilising a variety of strategies to obtain their current distributions (Väinölä et al. 2007; Wilson 2007; Poore and Bruce 2012). This broad biogeographic/evolutionary history can allow investigators to infer a great deal of information through phylogenetic reconstruction, not only regarding the organisms themselves but also the systems they occupy.

Traditional methods of phylogenetic analysis, such as the use of morphological characters, have proven insufficient for discriminating taxa within the Peracarida. Members of the order Amphipoda are especially troublesome as many species show variable morphology which makes analysis of higher‐level relationships difficult; similar patterns are also observed in other peracarid orders and lower‐level taxa (Spears et al. 2005; Wilson 2009; Sharma et al. 2021). Phylogenetic analyses incorporating molecular data offer powerful insights into evolutionary relationships and have revealed more intricate relationships within and among peracarids than were previously inferred (Kakui et al. 2011; Dimitriou et al. 2019; Cannizzaro et al. 2020). Such studies now form the backbone of a majority of modern phylogenetic and systematic analyses. However, molecular genetic analyses are often limited by the number of genes that can be sequenced; consequently, for a large number of understudied organisms or taxa from remote regions, many studies rely on a single gene or a handful of genes. This is especially true among invertebrates, for which a considerable number of species have yet to be sequenced. While such analyses have generated incredibly useful data, many of them have limited inferential power because they generate individual gene trees rather than more robust species trees. Such analyses are often hindered by discordance among genes, resulting in poor measures of support. While such hindrances can be minimised by inclusion of additional data, studies utilising single‐gene Sanger sequencing become logistically challenging as the number of genes increases.

Next‐generation sequencing (NGS) methodologies have greatly expanded the ability to investigate evolutionary relationships among organisms, allowing researchers to increase sampling of the genome and reconstruct more robust species trees. Cost‐effective genomic techniques such as reduced representation sequencing (RRS) target specific regions of the genome, decreasing cost while still examining data at a ‘genomic scale.’ Techniques such as restriction site‐associated DNA (RAD) sequencing utilise restriction sites or size selection to sequence homologous loci, which often show great utility for shallow‐level phylogenetics, population genetic analyses and genotyping of individuals (McCormack et al. 2013; Andrews et al. 2016). In a similar fashion, other target enrichment/sequence capture methods such as anchored hybrid enrichment and ultraconserved elements also target specific loci throughout the genome, but with the ability to resolve differences at deeper taxonomic levels (McCormack et al. 2013; Faircloth 2017).

Ultraconserved elements (UCEs) are regions of an organism's genome that are shared among taxa across large evolutionary distances; because they are ‘conserved’ in an evolutionary sense, these elements allow the examination of deep relationships between taxa; at the same time, they are bordered by more variable flanking regions which allow for the examination of shallower‐level relationships as well (Faircloth et al. 2012; Faircloth 2017). These features grant UCEs considerable phylogenetic utility, allowing researchers to address hypotheses at multiple taxonomic levels; recent targeted enrichment studies utilising UCEs have demonstrated their value at both deep (within family or genus) and shallow (population) levels (Faircloth 2017; Starrett et al. 2016; Winker et al. 2018). A number of such studies have targeted arthropods and developed generalised probe sets for taxa such as the Hymenoptera (Branstetter et al. 2017), Diptera (Faircloth 2017), Hemiptera (Faircloth 2017), Coleoptera (Faircloth 2017; Baca et al. 2017) and Arachnida (Faircloth 2017; Starrett et al. 2016). To date, only a single probe set has been developed solely for non‐insect crustaceans (Geburzi et al. 2024). Furthermore, only a handful of studies have targeted crustaceans (primarily Decapoda) using phylogenomic methodologies of any sort (Wolfe et al. 2019; Glon et al. 2022). Given the diversity present within the Crustacea, additional tools for generating phylogenomic data are likely to offer great benefit for elucidating their fascinating evolutionary and biogeography histories.

In this study, we introduce a probe set to target ultraconserved elements for the superorder Peracarida, with a focus on the Amphipoda and Isopoda, which together contain more than 95% of described peracarid species and are also located on different branches of the peracarid phylogenetic tree (Höpel et al. 2022). Published genome assemblies from several amphipod and isopod families, representing a majority of currently sequenced peracarid genomes at the time of design, were utilised to develop a probe set that would function at similar levels across various amphipod and isopod taxa and, likely, the remainder of the order. UCE probes were tested in silico and upon verification were then tested in vitro with representatives of the amphipod families Hyalellidae and Crangonyctidae, and the isopod family Sphaeromatidae. These families are among the most speciose and widespread in their respective orders; furthermore, the Crangonyctidae is an ancient and taxonomically difficult family making it an ideal candidate for such an analysis (Copilaş‐Ciocianu et al. 2019). Given the taxonomic breadth of our investigation, this probe set should prove useful for genomic analysis across the Peracarida.

## 2 Methods

### 2.1 Probe Design and UCE Identification

Genome scaffold assemblies for 13 species were downloaded from NCBI's genome assembly database (http://ncbi.nlm.nih.gov/assembly/; Table 1). These assemblies represented the majority of sequenced peracarid genomes at the time of probe design; they were used in both probe design and in silico testing. Selected genomes included representatives of four amphipod families, five isopod families, one mysid family and a single decapod family that was used as an outgroup (Table 1).

**TABLE 1:** Genome assemblies used for UCE probe design, along with the number of scaffolds for each assembly.

Order	Family	Species	Accession	Scaffolds	LE +10	LE +11	LE +12	LRT	Coleoptera	Decapoda
Amphipoda	Hyalellidae	*Hyalella* cf. *azteca*	GCF_000764305.1	17,396	7965	4208	5000	4190	38	194
Amphipoda	Hyalidae	*Parhyale hawaiensis*	GCA_001587735.2	278,189	7375	4399	4795	4339	39	176
Amphipoda	Talitridae	*Platorchestia* sp.	GCA_014220935.1	39,873	8652	4354	5153	4350	38	223
Amphipoda	Talitridae	*Speziorchestia grillus*	GCA_014899125.1	143,039	8549	4362	5124	4362	33	223
Amphipoda	Talitridae	*Trinorchestia longiramus*	GCA_006783055.1	30,897	8258	4284	5027	4258	34	204
Amphipoda	Gammaridae	*Gammarus roeselii*	GCA_016164225.1	1,130,582	7805	4149	4902	4127	33	213
Mysida	Mysidae	*Archaeomysis grebnitzkii*	GCA_031471125.1	373,499	7827	4394	4689	3515	46	188
Isopoda	Trachelipodidae	*Trachelipus rathkii*	GCA_015478945.1	239,756	5739	3535	3439	2294	39	106
Isopoda	Armadillidiidae	*Armadillidium nasatum*	GCA_009176605.1	25,196	7974	4367	4539	2420	32	130
Isopoda	Ligiidae	*Ligia exotica*	GCA_002091915.1	1,663,556	7587	3960	4252	3932	37	152
Isopoda	Idoteidae	*Idotea baltica*	GCA_023373965.1	338,085	7731	4040	3976	4039	40	171
Isopoda	Cirolanidae	*Bathynomus jamesi*	GCA_023014485.1	22,825	7142	3825	4263	3804	28	142
Decapoda	Palaemonidae	*Palaemon carinicauda*	GCA_004011675.1	9,470,451	4369	3107	3125	2804	32	504
				Averages	7459	4076	4483	3726	36	202

*Note:* The number of loci extracted (LE) in silico from varying exemplar counts during the probe design using *Parhyale hawaiensis* and the number of loci retained (LRT) from the +11 *P. hawaiensis* probe set analysis are also given. In addition, loci retained (LRT) from the analysis of the coleopteran and decapod‐specific probe sets are also presented here.

The open‐source program PHYLUCE v1.7.1 (Faircloth 2016) was used for both probe design and the identification of UCE loci, following the detailed methodology set forth by Faircloth (2017). Genomic sequence data were converted into 2bit files using faToTwoBit from the BLAT suite (Kent 2002). Using ART ILLUMINA (Huang et al. 2012), 100‐bp paired‐end reads were simulated from each genome without sequencing error, and alignments of simulated reads from remaining non‐outgroup taxa were generated (Table 2). We conducted a ‘base genome test’ (Gustafson et al. 2019) to maximise the number of UCE loci recovered during probe design by running the bait design/UCE loci identification protocols in parallel utilising several different base genomes. Eight taxa were selected to serve as ‘base genomes’ with the following probe/bait design and in silico steps being run separately with each taxon (Table 2). Stampy v1 (Lunter and Goodson 2011) was used to map conserved regions of each read‐simulated genome to the base genome, with a substitution rate set at 0.05; these resulting alignments were converted into BAM format, using SAMTOOLS (Li et al. 2009) and then into a BED format using BEDtools (Quinlan and Hall 2010). BED files were sorted by scaffold/contig and by position along scaffold/contig; alignment positions that were close (< 100 bp) were then merged using BEDtools. Additionally, sequences that included > 25% masked regions, ambiguous bases or regions that were < 80 bp were removed using the *phyluce_probe_strip_masked_loci_from_set* command in PHYLUCE. The result was BED files containing regions of conserved sequences shared between the base taxon and the exemplar taxa. Shared conserved loci were determined using the *phyluce_probe_query_multi_merge_table* command in PHYLUCE with a single exemplar taxon selected. An SQLite database was created for up to 528,558 loci across all base and exemplar taxa (Table 2). From these loci, regions up to 160 bp were extracted from the base genome using the *phyluce_probe_get_genome_sequences_from_bed* command in PHYLUCE. An additional filter was utilised to remove masked regions (> 25%), ambiguous bases or regions that were too short (< 80 bp); the remaining loci were then used to design temporary probe sets. Probes of 120 bp were tiled such that each locus was overlapped in the middle by 40 bp (3× density). This temporary bait set was then screened to remove probes with > 25% masked bases or high (> 70%)/low (< 30%) GC content. Potential duplicates were also removed using the *phyluce_probe_remove_duplicate_hits_from_probes_using_lastz* command in PHYLUCE. These temporary probe sets (up to 608,971 probes targeting up to 472,338 loci; Table 2) were aligned back to their base and exemplar genomes with an identity value of 50%. From these alignments, probes that matched multiple loci were removed. From the alignment files, loci were buffered to 180 bp and sliced from the exemplar genomes. To test the effects of varying numbers of exemplar genomes further downstream in the pipeline, an additional test was run selecting varying numbers (+2, +10, +11, +12) of exemplar taxa for all of the base genomes tested (Table 2). Of these, the +2 test was run using a more limited number of exemplar taxa, while the other tests were all run under the same parameters described here. For the four exemplar taxa options, SQLite databases were created for targeted loci using the *phyluce_probe_query_multi_fasta_table* command in PHYLUCE. From these data, bait sets were designed to target these loci; 120 bp probes were tiled (3× density, middle overlap) and screened for high/low GC content, masked bases and duplicates. These ‘final’ bait sets included up to 193,019 non‐duplicated probes targeting up to 10,328 loci (Table 2).

**TABLE 2:** Results of base genome tests using several amphipod/isopod taxa and varying exemplar taxa.

Base	SQDB loci	TPS loci	TPS probes	ET	Final probes	Final loci	ISU
Amphipoda							
*Hyalella* cf. azteca*	225,439	2153	4283	2	16,174	890	869
*Hyalella* cf. *azteca*	212,665	200,512	227,587	10	183,471	9443	8555
*Hyalella* cf. *azteca*				11	114,239	5423	4156
*Hyalella* cf. *azteca*				12	53,339	2332	2212
Speziorchestia grillus*	480,310	2203	4280	2	18,114	1013	966
*Speziorchestia grillus*	463,003	433,704	608,971	10	189,562	9716	8856
*Speziorchestia grillus*				11	115,058	5466	5041
*Speziorchestia grillus*				12	52,118	2257	2158
Parhyale hawaiensis*	290,500	1808	3517	2	15,722	916	854
*Parhyale hawaiensis*	265,171	241,459	191,602	10	190,959	9894	8911
*Parhyale hawaiensis*				11	118,930	5665	5233
*Parhyale hawaiensis*				12	56,080	2.419	2328
*Platorchestia* sp.*	528,558	2443	4669	2	19,695	1084	1053
*Platorchestia* sp.	506,010	472,338	597,441	10	191,959	9855	8965
*Platorchestia* sp.				11	117,247	5532	4802
*Platorchestia* sp.				12	52,122	2272	2161
*Trinorchestia longiramus*	491,162	461,440	607,982	10	192,656	10,034	8994
*Trinorchestia longiramus*				11	120,645	5760	5216
*Trinorchestia longiramus*				12	54,274	2404	2253
Isopoda							
Armadillidium nasatum*	137,804	731	1449	2	3510	191	187
*Armadillidium nasatum*	295,775	247,609	157,640	10	159,447	8608	7751
*Armadillidium nasatum*				11	123,752	6221	5118
*Armadillidium nasatum*				12	81,614	3821	3576
Idotea baltica*	149,704	1194	2378	2	10,539	602	571
*Idotea baltica*	121,316	109,467	108,598	10	193,019	10,328	9359
*Idotea baltica*				11	151,908	7642	5351
*Idotea baltica*				12	102,226	4750	4478
*Ligia exotica*	128,277	86,415	102,383	10	172,513	9328	8359
*Ligia exotica*				11	134,686	6823	5215
*Ligia exotica*				12	92,756	4353	4062

*Note:* Differences in recovered loci observed during both the probe design stage and in silico analyses are indicated. Asterisks indicate analyses run with a smaller pool of exemplar taxa.

Abbreviations: ET, exemplar taxa; ISU, in silico unique loci; SQDB, SQLite database; TPS, temporary probe set.

### 2.2 In Silico Test

The performance of designed probes in aligning to existing genomes was tested in silico by following the PHYLUCE workflow. The *phyluce_probe_run_multiple_lastz_sqlite* command was used to align the UCE probes to 2bit formatted exemplar genomes and an outgroup genome ( *Palaemon carinicauda* ), with an identity value of 50%. For each probe test, the matching FASTA data were sliced out of each genome, plus 400 bp of 5′/3′ flanking region, using the *phyluce_probe_slice_sequence_from_genomes* command. As a final screen for duplicates, loci were matched back to the baits using the *phyluce_assembly_match_contigs_to_probes* command, with a minimum coverage of 80% and minimum identity of 80%. In addition, to test the efficacy of the new probe sets, they were compared against previously generated sets for the Coleoptera (Faircloth 2017) and Decapoda (Geburzi et al. 2024), using the same commands (Table 1). For the exemplar taxa targeted here, filtering steps resulted in the recovery of up to 9359 unique loci (Tables 1 and 2). Each locus was exported into a FASTA file and aligned with MAFFT (Katoh et al. 2002) in PHYLUCE. The resulting FASTA files were trimmed internally using GBlocks (Castresana 2000; Talavera and Castresana 2007) under default parameters in PHYLUCE, and a 70% complete matrix was assembled for each alignment in PHYLUCE. Maximum‐likelihood (ML) inference was then conducted using these matrices with IQTREE 2.2 (Minh et al. 2020), which was run using the ModelFinder algorithm to select the best‐fitting substitution models for each UCE locus, which were then analysed under an edge‐linked model (Chernomor et al. 2016; Kalyaanamoorthy et al. 2017). Statistical support was estimated using 1000 ultrafast bootstrap replicates (Minh et al. 2013) and the Shimodaira–Hasegawa approximate likelihood ratio test (Shimodaria and Hasegawa 1999; Guindon et al. 2010).

### 2.3 DNA Extraction and Target Enrichment

Individuals of the amphipod families Hyalellidae, Crangonyctidae and Gammaridae, plus isopods of the family Sphaeromatidae, were selected to test the designed probes in vitro; in total 20 individuals from 7 genera were targeted for analysis (Tables 3 and S1). Genomic DNA was extracted using a bead‐based protocol (Tin et al. 2014) or using Tissue & Insect DNA MiniPrep kits (Zymo Research) utilising a modified protocol (Cannizzaro et al. 2020). DNA concentration was quantified using Qubit fluorometry (Thermo Fisher Scientific), and quality was verified by running samples on a high‐weight 1% agarose gel. Individuals for which extraction yield was < 10 ng/μL were subjected to whole genome amplification (WGA) in order to increase concentration/yield for downstream sequencing; WGA was conducted using GenomiPhi V2 DNA Amplification kits (Cytiva Life Sciences) following the manufacturer's protocol. Target enrichment/sequencing was performed using the ‘+11’ probe set with *Parhyale hawaiensis* as a base genome. This probe set was synthesised by RAPiD Genomics (www.rapid‐genomics.com/), which also prepared libraries, enriched targets and performed Illumina sequencing using the company's ‘Capture‐Seq’ platform, following standard protocols.

**TABLE 3:** Contig and locus information generated from assembled sequence reads from the in vitro analysis of amphipod/isopod taxa.

ID	Species	# contigs	LE (+10)	LE (+12)	LE (+11)	Total bp	MLL (bp)	LR	LRT
MPJ‐2.1	*Thermosphaeroma thermophilum*	592,929	2252	1265	1133	3,275,345	1454.41	213	2039
T011	*Thermosphaeroma smithi*	559,294	1117	630	574	626,906	561.24	64	1053
T023	*Thermosphaeroma subequalum* (BQ)	703,943	1363	787	694	2,220,448	1629.08	154	1209
T059	*Thermosphaeroma macrura*	1,171,484	1853	1048	931	1157,756	624.8	147	1706
Th_spp_029	*Thermosphaeroma subequalum* (HSM)	578,577	2104	1183	1051	3,394,906	1613.54	251	1853
Th_spp_047	*Thermosphaeroma subequalum* (RGV)	635,161	1993	1092	997	2,366,425	1187.36	176	1817
Th_spp_056	*Thermosphaeroma subequalum* (LM)	256,990	1293	711	619	2,429,324	1878.82	158	1135
Th_spp_058	*Thermosphaeroma subequalum* (PR)	773,974	1269	699	615	1,480,926	1167.00	115	1154
AGC‐709.1	*Cassidinea ovalis*	268,952	637	346	319	261,551	410.59	50	487
AGC‐379.2	*Hyalella cretae*	593,483	4278	2523	2207	4,972,011	1162.22	662	3616
AGC‐388.3	*Hyalella cretae*	266,133	4558	2771	2431	11,128,937	2441.62	1410	3148
AGC‐436.2	*Hyalella muerta*	730,208	4767	2827	2508	9,160,738	1921.69	1270	3497
AGC‐437.1	*Hyalella sandra*	59,828	1507	938	846	3,320,411	2203.32	488	1019
AGC‐986.1	*Hyalella wakulla*	708,162	4864	2892	2485	4,601,941	946.12	819	4045
AGC‐192.1	Bactrurus mucronatus*	67,795	333	254	221	184,827	555.03	162	171
AGC‐592.1	*Stygobromus tenuis*	563,949	502	315	303	497,137	990.31	36	466
AGC‐524.3	*Crangonyx cornutus*	1,011,471	1866	1126	980	833,826	446.85	78	1788
AGC‐601.1	*Crangonyx bousfieldi*	451,878	635	349	335	216,315	340.65	28	607
AGC‐181.2	Gammarus balmorhea*	45,464	381	295	273	302,353	793.57	315	66
AGC_354.3	Gammarus lacustris*	48,508	384	312	283	317,563	826.98	190	194

*Note:* Asterisks indicate individuals sequenced under the +2 probe *P. hawaiensis* set; all other individuals were sequenced under the +11 *P. hawaiensis* probe set. Results for the number of loci extracted using varying sets during the UCE extraction stage are given, along with the number of loci retained for the final dataset (+10).

Abbreviations: LE, loci extracted; LR, loci removed (+10 *P. hawaiensis* ); LRT, loci retained (+10 *P. hawaiensis* ); MLL, mean (UCE) locus length in bp.

### 2.4 Probe Set Verification and In Vitro Analyses

Demultiplexed Illumina reads were processed using PHYLUCE v1.7.1 following the workflow outlined in the online phylogenomic pipeline. Reads were trimmed to remove adapters and low‐quality bases using Illumiprocessor with standard options (Faircloth 2013). To complement individuals sequenced here, read data from an additional ten individuals were downloaded from the NCBI Sequence Read Archive (SRA; www.ncbi.nlm.gov/sra), including eight additional amphipods/isopods and members of two other peracarid orders (Table S2). Reads were assembled using Spades 3.15 (Bankevich et al. 2012) within PHYLUCE. The *phyluce_assembly_match_contigs_to_probes* command was used to match probes to assemblies; the commands *phyluce_assembly_get_match_counts* and *phyluce_assembly_get_fastas_from_match_counts* were used to identify loci with minimum identity and coverage set to 60% and then extract/export them into a FASTA file. Due to the degree of relatedness of taxa within the dataset, loci were internally trimmed using GBlocks and then aligned utilising the *phyluce_align_seqcap_align* command. Data matrices of locus alignments were generated for a 60% complete data matrix in PHYLUCE. The preceding steps were performed utilising the sequencing probe set (+11, *P. hawaiensis* ), along with both the (+10, *P. hawaiensis* ) and (+12, *P. hawaiensis* ) probe sets, which were run independently in order to discern differences in UCE yield. Uncorrected *p‐*distances were calculated from the 60% matrix using the R package *ape* (Paradis et al. 2004), under a Kimura 1980 (K80) model and with pairwise deletions removed. Maximum‐likelihood inference was conducted using these matrices and IQTREE 2.2, which was run using the MODELFINDER algorithm to select best‐fitting substitution models for each UCE locus; these were analysed under an edge‐linked model. Statistical support was estimated using 1000 ultrafast bootstrap replicates and the Shimodaira–Hasegawa approximate likelihood ratio test.

In order to gauge the utility of the designed probe set at a population level, individuals from five populations of the sphaeromatid isopod *Thermosphaeroma subequalum* were sequenced. Sequences were imported into R and binary single nucleotide polymorphisms (SNPs) were extracted using the *fasta2genlight* function in the *adegenet* package (Jombart 2008). After conversion, SNP matrices were filtered to remove ambiguities and retain variable sites with < 25% missing data. Principal component analysis (PCA) was performed in R using the *glpca* function in the *adegenet* package; results were plotted using the R package *ggplot2* (Wilkinson 2011), based on the first two principal components.

## 3 Results

### 3.1 Base Genome Test and Probe Set

**FIGURE 1:**
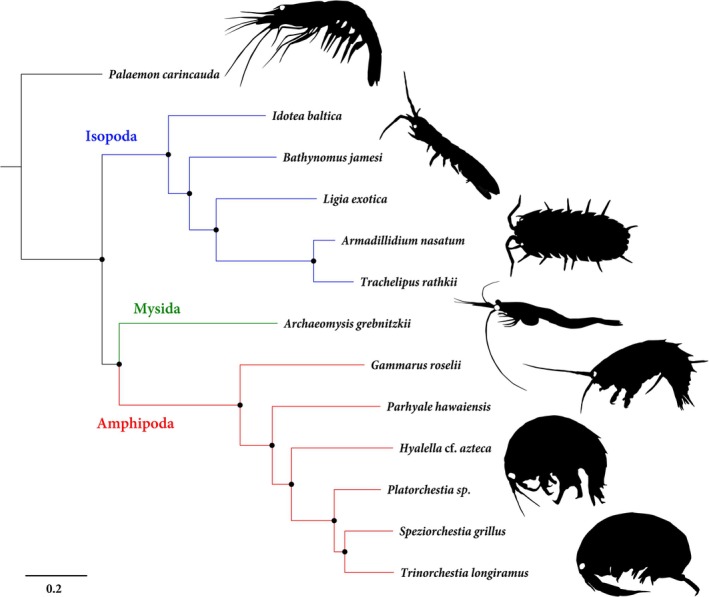
Maximum‐likelihood phylogeny reconstructed from in silico analysis, based on a 5233 locus (1,971,291 bp) concatenated dataset from a 70% complete matrix. Black circles represent node support with Ultrafast Bootstrap/Shimodaira–Hasegawa approximate likelihood ratio test values of 100.

Results of varying exemplar counts during the base genome tests demonstrated that all analyses run under the +10 parameter, regardless of the base genome selected, outperformed other analyses run under other parameters (+2, +11, +12; Table 2). Probe sets generated using the +10 parameter targeted an average of 9651 unique UCE loci and retained 8719 in silico, considerably more loci than the average of 5017 (+11) or 2904 (+12) retained in silico under other sets (Tables 1 and 2). However, probe sets designed under the +10 parameter required substantially more probes (159,000–193,000) versus the other newly designed sets (50,000–151,000; Table 2). The abundance of probes needed for the former made enrichment/sequencing logistically and financially difficult and as a result, probe sets under the next‐best performing parameter (+11) were considered for synthesis. Among these (+11) sets, an average of 4420 unique loci were identified with an average of 3948 retained in silico (Table 2). Of the 8 taxa selected to serve as base genomes, *Parhyale hawaiensis* performed the best in terms of loci retained in silico with 5233 of its 5665 unique loci retained under the (+11) probe set, followed closely by *Trinorchestia longiramus, Idotea baltica* and *Armadillidium nasatum* (Table 2). These sets recovered considerably more loci when compared to sets produced by Faircloth (2017) and Geburzi et al. (2024), which recovered 28–46 and 106–504 unique loci for the same taxa, respectively. As a result, the *P. hawaiensis* +11 probe set (containing 151,908 probes targeting 7642 unique UCE loci) was selected for synthesis and use in enrichment/sequencing and downstream phylogenomic analyses. Using this set, a 70% matrix was generated which included 5233 loci with a total length of 1,971,291 bp and 834,031 informative sites. The phylogeny reconstructed was highly supported at all nodes using both ultrafast bootstrapping (UFBS) and Shimodaira–Hasegawa approximate likelihood ratio tests (Figure 1).

### 3.2 In Vitro Analysis

**FIGURE 2:**
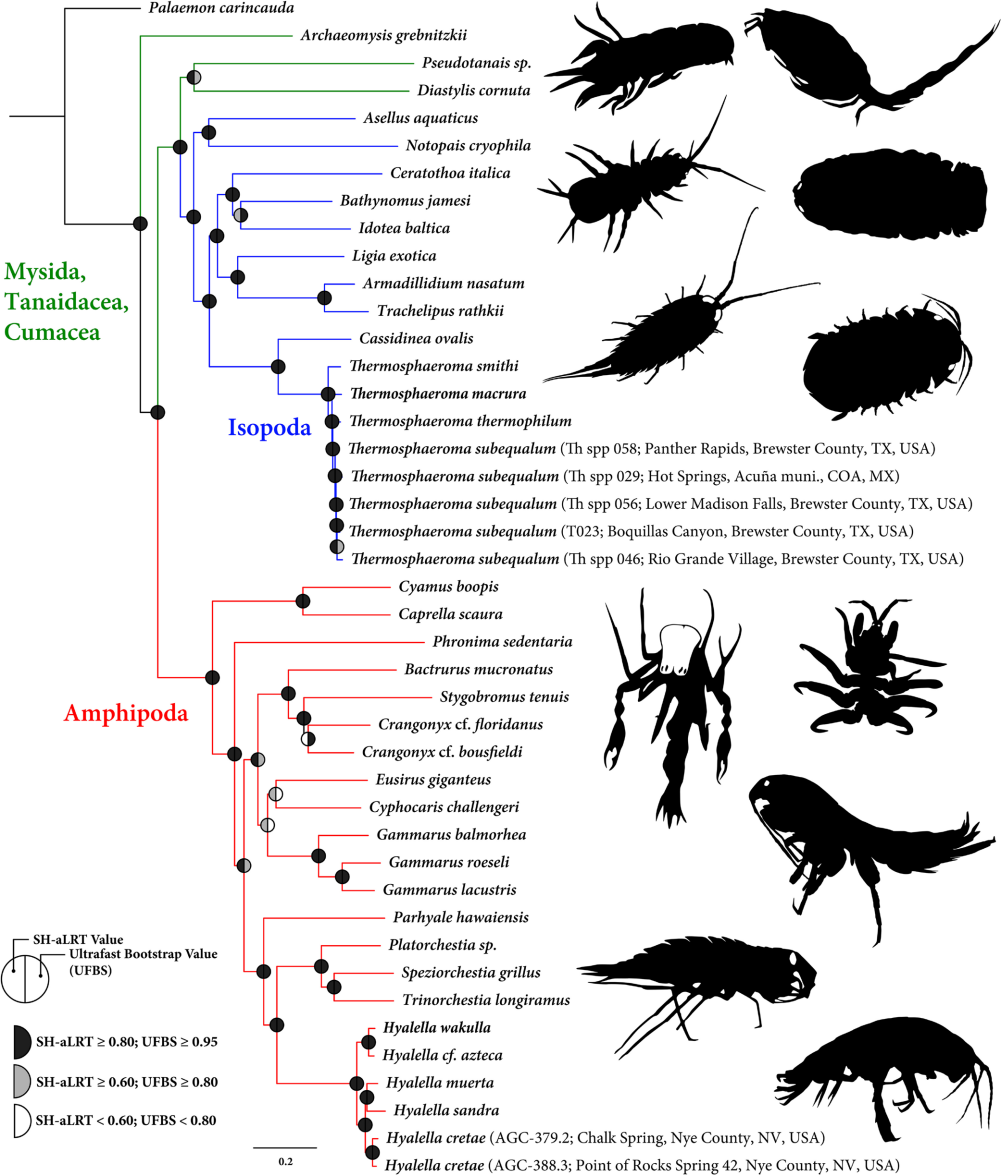
Maximum‐likelihood phylogeny reconstructed from in vitro analysis, based on a 616 locus (241,598 bp) concatenated dataset from a 60% complete matrix. Node support using two methodologies is indicated by shaded circles placed at nodes. SH‐aLRT, Shimodaira–Hasegawa approximate likelihood ratio test; UFBS, ultrafast bootstrap.

**FIGURE 3:**
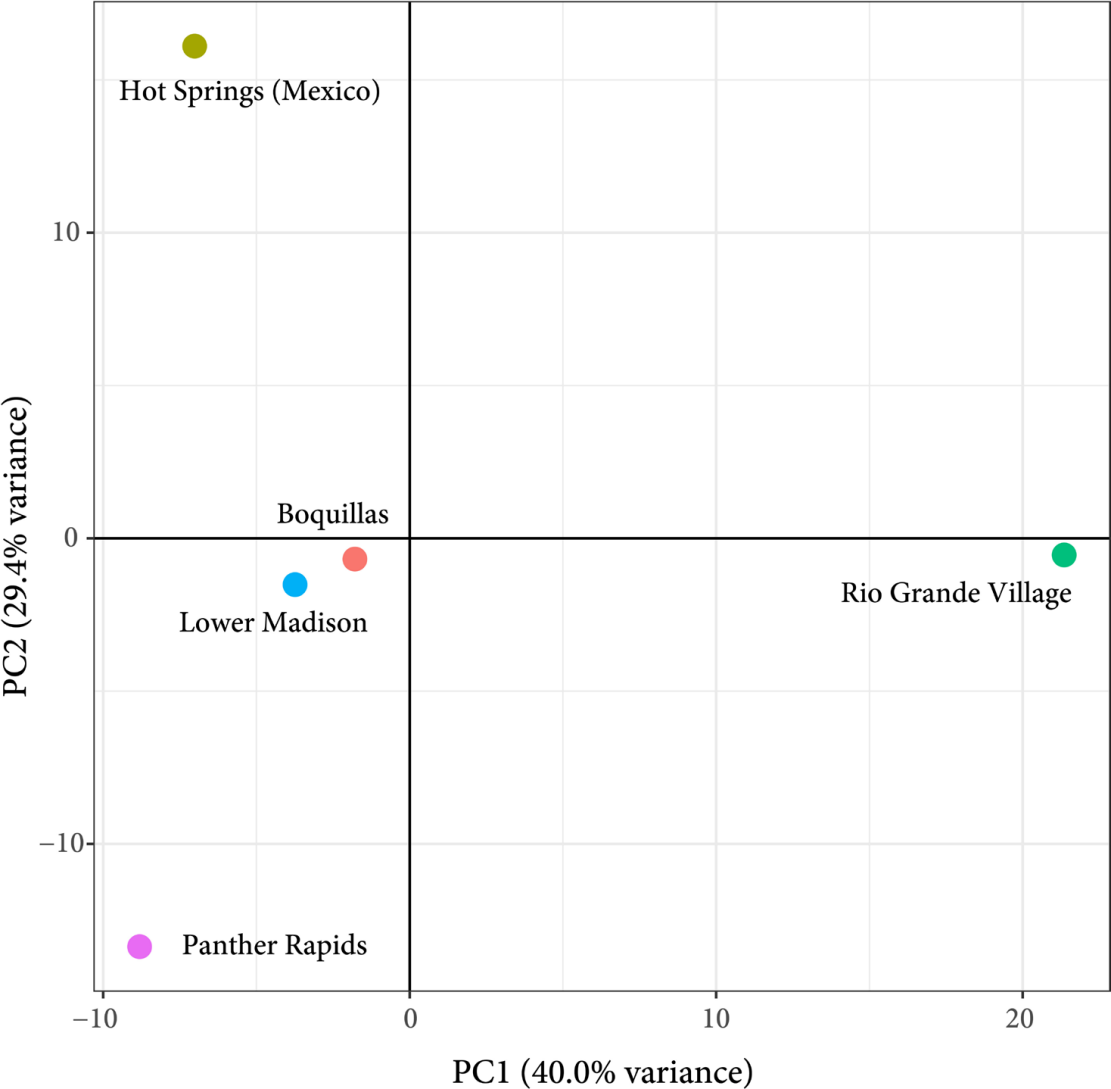
Principal component analysis of SNPs recovered from individuals of *Thermosphaeroma*.

Illumina reads from the 20 individuals sequenced ranged from 2,268,201 to 17,322,607 (mean: 12,030,386). The presence of low‐quality reads and adapters led to the removal of 0.33%–19.24% (mean: 5.8%) of reads per sample. Trimmed reads were assembled into 45,464 to 1,171,484 contigs (mean: 504,409) per individual with an average length of 1157 bp (Table 3). The dataset contained 9359 total UCE loci for all individuals, with 9122 UCE loci in the incomplete matrix. From the individuals newly sequenced, 28–1410 (mean: 340) loci were removed after filtering for duplicates (Table 3). The 60% complete data matrix retained 616 loci, with a total length of 241,598 bp and 149,233 informative sites. Maximum‐likelihood analyses of this matrix resulted in the reconstruction of a well‐supported phylogeny for all taxa included, with a majority of nodes possessing both UFBS/SHaLRT values > 0.95 (Figure 2). Uncorrected *p‐*distances calculated for members of the genus *Hyalella* based on the 70% matrix ranged from 0.01 to 0.05 (mean: 0.04). Individuals of two populations of *H. cretae* showed a considerably lower value at 0.007 (Table 4). Similar values were observed among species of *Thermosphaeroma* (0.03–0.06, mean: 0.05); populations of *T. subequalum* ranged slightly lower on average (0.01–0.03, mean: 0.03; Table 5). SNP screening of 5 individuals representing separate populations of *Thermosphaeroma subequalum* recovered 258,013 SNPs, with 1890 SNPs retained post‐filtering. Multivariate analysis of these SNPs suggests structuring and divergence among these populations, excluding Boquillas Canyon and Lower Madison Falls, with individuals clustering in a similar fashion to that observed in the maximum‐likelihood tree (Figure 3).

**TABLE 4:** Uncorrected *p‐*distances calculated for members of the genus *Hyalella* based on the 70% in vitro matrix.

—	*H. wakulla*	*H*. cf. *azteca*	*H. sandra*	*H. muerta*	*H. cretae* (CS)	*H. cretae* (POR)
*H. wakulla*	—	0.015	0.064	0.051	0.047	0.047
*H*. cf. *azteca*	0.015	—	0.060	0.049	0.046	0.046
*H. sandra*	0.064	0.060	—	0.050	0.042	0.044
*H. muerta*	0.033	0.049	0.050	—	0.051	0.031
*H. cretae* (CS)	0.047	0.046	0.042	0.033	—	0.008
*H. cretae* (POR)	0.047	0.046	0.044	0.031	0.008	—

Abbreviations: CS, Chalk Spring; POR, Points of Rocks Spring 42.

**TABLE 5:** Uncorrected *p‐*distances calculated for members of the isopod family Sphaeromatidae (genera *Cassidinidea* and *Thermosphaeroma*) based on the 70% in vitro matrix.

—	*C. ovalis*	*T. thermophilium*	*T. smithi*	*T. macrura*	BQC	PRS	RGV	HSM	LMF
*C. ovalis*	—	0.23	0.24	0.20	0.23	0.23	0.24	0.27	0.25
*T. thermophilium*	0.23	—	0.06	0.04	0.05	0.05	0.05	0.05	0.04
*T. smithi*	0.24	0.06	—	0.04	0.05	0.04	0.04	0.05	0.05
*T. macrura*	0.20	0.04	0.04	—	0.03	0.03	0.04	0.04	0.04
*T. subequalum* (BQC)	0.23	0.05	0.05	0.03	—	0.02	0.03	0.03	0.02
*T. subequalum* (PRS)	0.23	0.05	0.04	0.03	0.02	—	0.03	0.03	0.02
*T. subequalum* (RGV)	0.24	0.05	0.04	0.04	0.03	0.03	—	0.03	0.03
*T. subequalum* (HSM)	0.27	0.05	0.05	0.04	0.03	0.03	0.03	—	0.02
*T. subequalum* (LMF)	0.25	0.04	0.05	0.04	0.02	0.02	0.03	0.02	—

Abbreviations: BQC, Boquillas Canyon; HSM, Hot Springs, Mexico; LM, Lower Madison Falls; PRS, Panther Rapids; RGC, Rio Grande Village.

## 4 Discussion

### 4.1 Probe Set and In Silico Analyses

While phylogenomic methods, especially those employing targeted enrichment, are becoming increasingly common in phylogenetic and systematic studies, such studies targeting Crustacea are rare. For example, numerous insect and chelicerate genomes have been sequenced and several studies have explored evolutionary relationships utilising genomic data (Baca et al. 2017; Faircloth 2017; Sharma et al. 2021). In contrast, the decapods—a crustacean taxon with a large amount of active research and significant economic value—include < 50 published genomes and have only been the targets of a handful of phylogenomic studies (Wolfe et al. 2019; Glon et al. 2022; Geburzi et al. 2024). Other crustacean groups such as the Peracarida are the subject of very active phylogenetic research efforts but have received relatively little attention at a genomic level. At the time of this study, < 15 peracarid genomes were sequenced and relatively few genomic studies had been conducted on representative taxa, with most published studies limited to examination of mitogenomes or transcriptomes (Naumenko et al. 2017; Höpel et al. 2022). Here we utilised phylogenomic resources to develop generalised probe sets targeting members of the Peracarida. These probe sets employ ~10,000–100,000 probes targeting up to 10,000 UCE loci for members of both the Isopoda and Amphipoda.

When the efficacy of our probe set was tested in silico using peracarid genomes, an average of 4483–7459 loci was recovered from the targeted taxa across all parameters, with an average of 3726 loci retained post‐filtering (Table 1). The numbers of loci targeted, recovered and extracted here are similar to those for other probe sets developed for the Arachnida and Caenogastropoda, each of which retained over 1000 loci post‐filtering (Faircloth 2017; Starrett et al. 2016; Goulding et al. 2023). In addition, our set was able to recover considerably more loci for members of the Peracarida versus other probe sets designed for non‐insect Crustacea. Sets produced by Faircloth (2017) for Coleoptera and by Geburzi et al. (2024) for Decapoda recovered an average of 36 and 202 unique loci respectively when tested in silico (Table 1). The loci both targeted and recovered by sets presented here represent an increase by an order of magnitude. In addition, the numbers of recovered loci in these new sets were similar across taxa, with amphipods, isopods and other peracarid orders displaying similar numbers of loci (Table 1; Table S2). Our results suggest that the probe set functions well for members of all of these orders and can be employed for studies targeting single or multiple groups of peracarids. In addition, while amphipods and isopods represent some of the most species‐rich orders within the Peracarida and appear similar, they are not sister orders, with other peracarid taxa such as the Mysidacea and Cumacea/Tanaidacea being recovered as sister to the Amphipoda and Isopoda respectively (Höpel et al. 2022). Our results corroborate this, placing the Cumacea/Tanaidacea as sister to the Isopoda (Figure 2) and the Mysida as either basally derived (Figure 1) or sister to the Amphipoda (Figure 2). This discordance may be due to the limited taxon sampling in our in silico analysis or the differing number of loci retained in both analyses (5233 vs. 616). Further, more‐focused genomic sampling would likely help elucidate this issue. Because our probe set was able to recover a significant number of UCE loci for both amphipods and isopods, which are representatives of different peracarid lineages, the set is likely to prove useful throughout the superorder and could be employed for studies targeting other peracarid taxa, including poorly studied orders such as the Ingolfiellidea and Thermosbaenacea.

The phylogenomic analyses we performed in silico placed all taxa with high measures of support, clearly separating both families and genera of Isopoda and Amphipoda (Figure 1). Molecular phylogenetic analyses of the Oniscidea have suggested that the suborder may be polyphyletic, with taxa such as *Ligia* showing more affinity to taxa from other suborders, particularly the sphaeromatidea (Dimitriou et al. 2019). Although isopod taxon sampling at the suborder level was limited, our phylogenomic analyses recovered a monophyletic Oniscidea, congruent with morphology and current taxonomy, with considerable distances observed between members of Crinocheta (*Armadillidum nasatum* + *Trachelipus rathkii* ) and Diplocheta ( *Ligia exotica* ; Figure 1). Amphipod taxa identified in silico primarily included members of the superfamilies Hyaloidea and Talitroidea; results generated from our phylogenomic analyses clarify relationships previously proposed in phylogenetic datasets. A biogeographic analysis of the superfamily Hyaloidea reported differences in the placement of the Hyalellidae and Talitroidea based on the analytical methods employed (Cannizzaro and Berg 2022). Maximum likelihood placed the Talitroidea in a clade with the Hyalellidae, while Bayesian Inference placed the Talitroidea basally derived to the superfamily (Cannizzaro and Berg 2022). Our results match the former hypothesis, supporting the Hyalidae as the more basally derived family (Figure 1). Although involving a limited number of taxa, the results generated by our in silico analyses demonstrate the utility of this probe set for investigating evolutionary and biogeographic hypotheses in both the Amphipoda and Isopoda. Additional analyses using this probe set to examine a wider range of taxa have the potential to resolve relationships across the Peracarida.

### 4.2 In Vitro Analyses

Our probe sets were used to sequence members of the amphipod families Gammaridae, Crangonyctidae and Hyalellidae, along with the isopod family Sphaeromatidae. These families were selected due to specimen availability, species richness and varying evolutionary histories; gammarids show relatively shallow Miocene diversification (Hou and Sket 2016), while crangonyctids/hyalellids diverged as far back as the Cretaceous (Copilaş‐Ciocianu et al. 2019; Cannizzaro and Berg 2022). In addition, a number of other families/genera from across the Peracarida were obtained from the NCBI Sequence Read Archive, filling in much of the Isopod/Amphipod phylogenies and allowing for members of the orders Cumacea and Tanaidacea to also be included (Table S2). These probe sets were successful in extracting 9359 unique loci from the taxa selected, with an average of 1897 loci among newly sequenced taxa pre‐filtering (Table 3). Phylogenetic trees generated using this dataset produced well‐supported topologies illustrating relationships among all the individuals sequenced (Figure 2).

In addition to demonstrating utility at levels above species, data generated here also display utility within species. Individuals from two populations of the amphipod *Hyalella cretae* were included here, the type locality (Chalk Spring) and the nearby Point of Rocks Spring #42 (POR); these were included as the specific status of the POR population was unclear when examined with Sanger loci which displayed high mitochondrial differentiation and low nuclear differentiation (Cannizzaro et al. 2023). However, our results indicate that these two populations are very closely related and likely conspecific, displaying an uncorrected *p‐*distance of 0.0078 which is almost an order of magnitude lower than the average of 0.04 we observed among other species (Table 4). In addition, five populations of the isopod *Thermosphaeroma subequalum* along a stretch of the Rio Grande were also included here to test the utility of the dataset within species. These individuals were clearly differentiated by our dataset, displaying uncorrected *p*‐distances of 0.01–0.03 (Table 5). Surprisingly, these distances were somewhat similar and overlapped with those (0.03–0.06, mean: 0.05) observed between other members of the genus *Thermospheroma*, suggesting deeper divergences which may rise to species level (Table 5). In addition, a total of 258,013 SNPs were recovered from these populations, with 1890 retained post‐filtering. Multivariate analysis of these SNPs demonstrated similar results, with most individuals not clustering closely in a PCA (Figure 3). Our results are concordant with those generated by Learned (2004) who examined the same populations using electrophoretic analysis and recovered similar significant genetic structure within the species, likely promoted by geographic isolation and the species' habitat preferences (thermal springs feeding into the Rio Grande).

## 5 Conclusions

We developed universal probe sets targeting members of the peracarid orders Isopoda and Amphipoda, utilising publicly available genomes from 12 peracarid species. In silico analyses of this probe set demonstrate its usefulness for isolating UCEs from amphipods, isopods and mysids. Results generated by the in silico analyses were bolstered by an in vitro analysis targeting members of the amphipod families Hyalellidae and Crangonyctidae and the isopod family Sphaeromatidae; the in vitro analysis was successful in isolating over 9000 unique UCE loci across the taxa sampled, of which over 600 were retained in a 60% matrix. Phylogenetic analyses performed using both the in silico and in vitro datasets resulted in trees with well‐supported nodes at both deep and shallow taxonomic levels. Furthermore, our data also displayed utility at levels below species, with phylogenetic signal observed among populations for both the amphipod *Hyalella cretae* and the isopod *Thermosphaeroma subequalum* . Our probe sets and the results of our analyses introduce genomic resources into peracarid research. Further analysis of peracarid taxa through a phylogenomic lens offers a great deal of opportunity, not only for improving phylogenies, but also for developing analytical methods that employ more robust data. In addition, higher‐throughput sequencing methodologies such as those employed here can be used for the sequencing of older and/or degraded DNA, which can allow individuals not easily sequenceable under Sanger methodologies to be included in phylogenetic analyses.

## Author Contributions

A.G.C. conceived the project, designed the probe set, analysed sequence data and drafted the paper. D.J.B. provided funding and reviewed the manuscript.

## Disclosure

Benefit‐sharing statement: The research in this publication complies with the relevant national laws implementing the Convention on Biological Diversity and the Nagoya Protocol.

## Conflicts of Interest

The authors declare no conflicts of interest.

## Supporting information


**TABLE S1:** men70078‐sup‐0001‐TableS1‐S2.pdf.

## Data Availability

Raw sequence reads are deposited in the NCBI SRA (BioProject PRJNA1344336). Aligned sequence files used here and the probe sets are available through Dryad (http://datadryad.org/share/HhONVRe4rn8wyyLTY_q13fxzMD0hA87hIt1xxpt_HnI).

## References

[men70078-bib-0001] Andrews, K. R. , J. M. Good , M. R. Miller , G. Luikart , and P. A. Hohenlohe . 2016. “Harnessing the Power of RADseq for Ecological and Evolutionary Genomics.” Nature Reviews Genetics 17: 81–92. 10.1038/nrg.2015.28 PMC4823021 26729255

[men70078-bib-0002] Arfianti, T. , and M. J. Costello . 2020. “Global Biogeography of Marine Amphipod Crustaceans: Latitude, Regionalization, and Beta Diversity.” Marine Ecology Progress Series 638: 83–94.

[men70078-bib-0003] Baca, S. M. , A. Alexander , G. T. Gustafson , and A. E. Z. Short . 2017. “Ultraconserved Elements Show Utility in Phylogenetic Inference of Adephaga (Coleoptera) and Suggest Paraphyly of ‘Hydradephaga’.” Systematic Entomology 42: 786–795.

[men70078-bib-0004] Bankevich, A. , S. Nurk , D. Antipov , et al. 2012. “SPAdes: A New Genome Assembly Algorithm and Its Applications to Single‐Cell Sequencing.” Journal of Computational Biology 19: 455–477. 22506599 10.1089/cmb.2012.0021 PMC3342519

[men70078-bib-0005] Branstetter, M. G. , J. T. Longino , P. S. Ward , and B. C. Faircloth . 2017. “Enriching the Ant Tree of Life: Enhanced UCE Bait Set for Genome‐Scale Phylogenetics of Ants and Other Hymenoptera.” Methods in Ecology and Evolution 8: 768–776.

[men70078-bib-0006] Cannizzaro, A. G. , and D. J. Berg . 2022. “Gone With Gondwana: Amphipod Diversification in Freshwaters Followed the Breakup of the Supercontinent.” Molecular Phylogenetics and Evolution 171: 107464. 35358695 10.1016/j.ympev.2022.107464

[men70078-bib-0007] Cannizzaro, A. G. , J. R. Gibson , and T. R. Sawicki . 2020. “A New Enigmatic Genus of Subterranean Amphipod (Amphipoda: Bogidielloidea) From Terrell County, Texas, With the Establishment of Parabogidiellidae, Fam. Nov., and Notes on the Family Bogidiellidae.” Invertebrate Systematics 34: 504–518.

[men70078-bib-0008] Cannizzaro, A. G. , C. J. Lange , and D. J. Berg . 2023. “A New Species of Stygobitic *Hyalella* Smith, 1874 (Amphipoda: Hyalellidae) From Ash Meadows National Wildlife Refuge, Nevada, USA, With Discussion of the Unique Presence of the Species in the Nearctic Groundwater Fauna.” Journal of Crustacean Biology 43: ruad073.

[men70078-bib-0009] Castresana, J. 2000. “Selection of Conserved Blocks From Multiple Alignments for Their Use in Phylogenetic Analyses.” Molecular Biology and Evolution 17: 540–552. 10742046 10.1093/oxfordjournals.molbev.a026334

[men70078-bib-0010] Chernomor, O. , A. von Haeseler , and B. Q. Minh . 2016. “Terrace Aware Data Structure for Phylogenomic Inference From Supermatricies.” Systematic Biology 65: 997–1008. 27121966 10.1093/sysbio/syw037 PMC5066062

[men70078-bib-0011] Copilaş‐Ciocianu, D. , Š. Borko , and C. Fišer . 2020. “The Late Blooming Amphipods: Global Change Promoted Post‐Jurassic Ecological Radiation Despite Paleozoic Origin.” Molecular Phylogenetics and Evolution 143: 106664. 31669816 10.1016/j.ympev.2019.106664

[men70078-bib-0012] Copilaş‐Ciocianu, D. , D. Sidorov , and A. Gontcharov . 2019. “Adrift Across Tectonic Plates: Molecular Phylogenetics Supports the Ancient Laurasian Origin of Old Limnic Crangonyctid Amphipods.” Organisms Diversity & Evolution 19: 191–207.

[men70078-bib-0013] Dimitriou, A. C. , S. Tati , and S. Sfenthourakis . 2019. “Genetic Evidence Against Monophyly of Oniscidea Implies a Need to Revise Scenarios for the Origin of Terrestrial Isopods.” Scientific Reports 9: 18508. 31811226 10.1038/s41598-019-55071-4 PMC6898597

[men70078-bib-0014] Faircloth, B. C. 2013. Illumiprocessor: A Trimmomatic Wrapper for Parallel Adapter and Quality Trimming. 10.6079/J9ILL.

[men70078-bib-0015] Faircloth, B. C. 2016. “PHYLUCE Is a Software Package for the Analysis of Conserved Genomic Loci.” Bioinformatics 32: 786–788. 26530724 10.1093/bioinformatics/btv646

[men70078-bib-0016] Faircloth, B. C. 2017. “Identifying Conserved Genomic Elements and Designing Universal Bait Sets to Enrich Them.” Methods in Ecology and Evolution 8: 1103–1112.

[men70078-bib-0017] Faircloth, B. C. , J. E. McCormack , N. G. Crawford , M. G. Harvey , R. T. Brumfield , and T. C. Glenn . 2012. “Ultraconserved Elements Anchor Thousands of Genetic Markers Spanning Multiple Evolutionary Time Scales.” Systematic Biology 61: 717–726. 22232343 10.1093/sysbio/sys004

[men70078-bib-0018] Geburzi, J. C. , P. C. Rodríguez‐Flores , S. Derkarabetian , and G. Giribet . 2024. “From the Shallows to the Depths: A New Probe Set to Target Ultraconserved Elements for Decapoda and Other Malacostraca.” Frontiers in Marine Science 11: 1429314.

[men70078-bib-0019] Glon, M. G. , M. B. Broe , K. A. Crandall , et al. 2022. “Anchored Hybrid Enrichment Resolves the Phylogeny of *Lacunicambarus* Hobbs, 1969 (Decapoda: Astacidea: Cambaridae).” Journal of Crustacean Biology 42: ruab073.

[men70078-bib-0020] Goulding, T. C. , E. E. Strong , and A. M. Quattrini . 2023. “Target‐Capture Probes for Phylogenomics of the Caenogastropoda.” Molecular Ecology Resources 23: 1372–1388. 36997300 10.1111/1755-0998.13793

[men70078-bib-0021] Guindon, S. , J. F. Dufayard , V. Lefort , M. Anisimova , W. Hordijk , and O. Gascuel . 2010. “New Algorithms and Methods to Estimate Maximum‐Likelihood Phylogenies: Assessing the Performance of PhyML 3.0.” Systematic Biology 59: 307–321. 20525638 10.1093/sysbio/syq010

[men70078-bib-0022] Gustafson, G. T. , A. Alexander , J. S. Sproul , J. M. Pflug , D. R. Maddison , and A. E. Short . 2019. “Ultraconserved Element (UCE) Probe Set Design: Base Genome and Initial Design Parameters Critical for Optimization.” Ecology and Evolution 9: 6933–6948. 31312430 10.1002/ece3.5260 PMC6617817

[men70078-bib-0023] Höpel, C. G. , D. Yeo , M. Grams , R. Meier , and S. Richter . 2022. “Mitogenomics Supports the Monophyly of Mysidacea and Peracarida (Malacostraca).” Zoologica Scripta 51: 603–613.

[men70078-bib-0024] Hou, Z. , and B. Sket . 2016. “A Review of Gammaridae (Crustacea: Amphipoda): The Family Extent, Its Evolutionary History, and Taxonomic Redefinition of Genera.” Zoological Journal of the Linnean Society 176: 323–348.

[men70078-bib-0025] Huang, W. , L. Li , J. R. Myers , and G. T. Marth . 2012. “ART: A Next‐Generation Sequencing Read Simulator.” Bioinformatics 28: 593–594. 22199392 10.1093/bioinformatics/btr708 PMC3278762

[men70078-bib-0026] Jombart, T. 2008. “Adegenet: A R Package for the Multivariate Analysis of Genetic Markers.” Bioinformatics 24: 1403–1405. 18397895 10.1093/bioinformatics/btn129

[men70078-bib-0027] Kakui, K. , T. Katoh , S. F. Hiruta , N. Kobayashi , and H. Kajihara . 2011. “Molecular Systematics of Tanaidacea (Crustacea: Peracarida) Based on 18S Sequence Data, With an Amendment of Suborder/Superfamily‐Level Classification.” Zoological Science 28: 557–749. 10.2108/zsj.28.749 21967223

[men70078-bib-0028] Kalyaanamoorthy, S. , B. Q. Minh , T. K. F. Wong , A. von Haeseler , and L. S. Jermiin . 2017. “ModelFinder: Fast Model Selection for Accurate Phylogenetic Estimates.” Nature Methods 14: 587–589. 28481363 10.1038/nmeth.4285 PMC5453245

[men70078-bib-0029] Katoh, K. , K. Misawa , K. I. Kuma , and T. Miyata . 2002. “MAFFT: A Novel Method for Rapid Multiple Sequence Alignment Based on Fast Fourier Transform.” Nucleic Acids Research 30: 3056–3066. 10.1093/nar/gkf436 PMC135756 12136088

[men70078-bib-0030] Kent, W. J. 2002. “BLAT—The BLAST‐Like Alignment Tool.” Genome Research 30: 3059–3066. 10.1101/gr.229202 PMC187518 11932250

[men70078-bib-0031] Learned, J. K. 2004. Genetic Differences Among Populations of the Freshwater Isopod Thermosphaeroma Subequalum with a Revised Generic Key (Doctoral dissertation). Northern Arizona University.

[men70078-bib-0032] Li, H. , B. Handsaker , A. Wyosker , et al. 2009. “The Sequence Alignment/Map Format and SAMtools.” Bioinformatics 25: 2078–2079. 19505943 10.1093/bioinformatics/btp352 PMC2723002

[men70078-bib-0033] Lunter, G. , and M. Goodson . 2011. “Stampy: A Statistical Algorithm for Sensitive and Fast Mapping of Illumina Sequence Reads.” Genome Research 21: 936–939. 20980556 10.1101/gr.111120.110 PMC3106326

[men70078-bib-0034] McCormack, J. E. , S. M. Hird , A. J. Zellmer , B. C. Carstens , and R. T. Brumfield . 2013. “Applications of Next‐Generation Sequencing to Phylogeography and Phylogenetics.” Molecular Phylogenetics and Evolution 66: 1189–1203. 10.1016/j.ympev.2011.12.007 22197804

[men70078-bib-0035] Minh, B. Q. , M. A. Nguyen , and A. von Haeseler . 2013. “Ultrafast Approximation for Phylogenetic Bootstrap.” Molecular Biology and Evolution 30: 1188–1195. 23418397 10.1093/molbev/mst024 PMC3670741

[men70078-bib-0036] Minh, B. Q. , H. A. Schmidt , O. Chernomor , et al. 2020. “IQ‐TREE 2: New Models and Efficient Methods for Phylogenetic Inference in the Genomic Era.” Molecular Biology and Evolution 37: 1530–1534. 32011700 10.1093/molbev/msaa015 PMC7182206

[men70078-bib-0037] Naumenko, S. A. , M. D. Logacheva , N. V. Popova , et al. 2017. “Transcriptome‐Based Phylogeny of Endemic Lake Baikal Amphipod Species Flock: Fast Speciation Accompanied by Frequent Episodes of Positive Selection.” Molecular Ecology 26: 536–553. 27859915 10.1111/mec.13927

[men70078-bib-0038] Paradis, E. , J. Claude , and K. Strimmer . 2004. “APE: Analyses of Phylogenetics and Evolution in R Language.” Bioinformatics 20: 289–290. 14734327 10.1093/bioinformatics/btg412

[men70078-bib-0039] Poore, G. C. B. 2005. “Peracarida: Monophyly, Relationships and Evolutionary Success.” Nauplius 13: 1–27.

[men70078-bib-0040] Poore, G. C. B. , and N. L. Bruce . 2012. “Global Diversity of Marine Isopods (Except Asellota and Crustacean Symbionts).” PLoS One 7: e43529. 22952700 10.1371/journal.pone.0043529 PMC3432053

[men70078-bib-0041] Quinlan, A. R. , and I. M. Hall . 2010. “BEDTools: A Flexible Suite of Utilities for Comparing Genomic Features.” Bioinformatics 26: 841–842. 20110278 10.1093/bioinformatics/btq033 PMC2832824

[men70078-bib-0042] Robin, R. , P. Gueriau , J. Luque , D. Jarvis , A. C. Daley , and R. Vonk . 2021. “The Oldest Peracarid Crustacean Reveals a Late Devonian Freshwater Colonization by Isopod Relatives.” Biology Letters 17: 20210226. 34129798 10.1098/rsbl.2021.0226 PMC8205522

[men70078-bib-0043] Sharma, P. P. , J. A. Ballesteros , and C. E. Santibáñez‐López . 2021. “What Is an ‘Arachnid’? Consensus, Consilience, and Confirmation Bias in the Phylogenetics of Chelicerata.” Diversity 13: 568.

[men70078-bib-0044] Shimodaria, H. , and M. Hasegawa . 1999. “Multiple Comparisons of Log‐Likelihoods With Applications to Phylogenetic Inference.” Molecular Biology and Evolution 16: 1114–1116.

[men70078-bib-0045] Spears, T. , R. W. DeBry , L. G. Abele , and K. Chodyla . 2005. “Peracarid Monophyly and Interordinal Phylogeny Inferred From Nuclear Small‐Subunit Ribosomal DNA Sequences (Crustacea: Malacostraca: Peracarida).” Proceedings of the Biological Society of Washington 118: 117–157.

[men70078-bib-0046] Starrett, J. , S. Derkarabetia , M. Hedin , R. W. Bryson Jr. , J. E. McCormack , and B. C. Faircloth . 2016. “High Phylogenetic Utility of an Ultraconserved Element Probe Set Designed for Arachnida.” Molecular Ecology Resources 17: 812–823. 27768256 10.1111/1755-0998.12621

[men70078-bib-0047] Talavera, G. , and J. Castresana . 2007. “Improvement of Phylogenies After Removing Divergent and Ambiguously Aligned Blocks From Protein Sequence Alignments.” Systematic Biology 56: 564–577. 17654362 10.1080/10635150701472164

[men70078-bib-0048] Tin, M. M.‐Y. , E. P. Economo , and A. S. Mikheyev . 2014. “Sequencing Degraded DNA From Non‐Destructively Sampled Museum Samples for RAD‐Tagging and Low‐Coverage Shotgun Phylogenetics.” PLoS One 9: e96793. 24828244 10.1371/journal.pone.0096793 PMC4020769

[men70078-bib-0049] Väinölä, R. , J. D. S. Witt , M. Grabowski , J. H. Bradbury , K. Jazdzewski , and B. Sket . 2007. “Global Diversity of Amphipods (Amphipoda: Crustacea) in Freshwater.” In Freshwater Animal Diversity Assessment, edited by E. V. Balian , C. Leveque , H. Segers , and K. Marten . Springer.

[men70078-bib-0050] Wilkinson, L. 2011. “ggplot2: Elegant Graphics for Data Analysis by Wickham, H.” Biometrics 67: 678–679.

[men70078-bib-0051] Wilson, G. D. F. 2007. “Global Diversity of Isopod Crustaceans (Crustacea; Isopoda) in Freshwater.” In Freshwater Animal Diversity Assessment, edited by E. V. Balian , C. Leveque , H. Segers , and K. Marten . Springer.

[men70078-bib-0052] Wilson, G. D. F. 2009. “The Phylogenetic Position of the Isopoda in the Peracarida (Crustacea: Malacostraca).” Arthropod Systematics & Phylogeny 67: 159–198.

[men70078-bib-0053] Winker, K. , T. C. Glenn , and B. C. Faircloth . 2018. “Ultraconserved Elements (UCEs) Illuminate the Population Genomics of a Recent, High‐Latitude Avian Speciation Event.” PeerJ 6: e5735. 30310754 10.7717/peerj.5735 PMC6174879

[men70078-bib-0054] Wolfe, J. M. , J. W. Breinholt , K. A. Crandall , et al. 2019. “A Phylogenomic Framework, Evolutionary Timeline and Genomic Resources for Comparative Studies of Decapod Crustaceans.” Proceedings of the Royal Society B 286: 20190079. 31014217 10.1098/rspb.2019.0079 PMC6501934

