# Supplementary material for: Brooding Phylogenomics: Target‐Capture Probe Sets for the Analysis of Ultraconserved Elements in the Peracarida
Source: Mol Ecol Resour. 2025 Nov 10;26(1):e70078. doi: 10.1111/1755-0998.70078 (PMC12627908; doi:10.1111/1755-0998.70078)
Supplement: Supplementary file 1 — TABLE S1: men70078‐sup‐0001‐TableS1‐S2.pdf. [file MEN-26-e70078-s001.pdf]

MOLECULAR ECOLOGY RESOURCES

Supplemental Information for:

Brooding phylogenomics: Target-capture probe sets for the analysis of ultraconserved elements in the Peracarida

Andrew G. Cannizzaro, David J. Berg

Table of Contents:

**Table S1**	Page 2
**Table S2**	Page 4

MOLECULAR ECOLOGY
RESOURCES

Table S1. Collection and extraction data for taxa sequenced for the *in-vitro* analysis. Dashes indicate localities for which the exact latitude/longitude could not be discerned**.**

Species	Cat. #	Locality	Latitude	Longitude	Collector(s)	Date Coll.	DNA concentration	WGA performed
Thermosphaeroma thermophilum	MPJ-2.1	Socorro Isopod Propagation Facility, Socorro County, NM, USA	34.0424	-106.93265	Mary P. Jones	Unknown	8.34 ng/μL	Y
Thermosphaeroma smithi	T011	Balneario San Diego, San Diego de Alcalá muni., CHH, MX	-	-	Unknown	Unknown	12.8 ng/μL	N
Thermosphaeroma subequalum	T023	Boquillas Canyon, Brewster County, TX, USA	-	-	Unknown	Unknown	0.28 ng/μL	Y
Thermosphaeroma macrura	T059	Julimes, CHH, MX	-	-	Unknown	Unknown	66.6 ng/μL	N
Thermosphaeroma subequalum	Th_spp_029	Hot Springs, Acuna municipality, COA, MX	-	-	Unknown	Unknown	2.9 ng/μL	Y
Thermosphaeroma subequalum	Th_spp_047	Rio Grande Village, Brewster County, TX, USA	-	-	Unknown	Unknown	10.8 ng/μL	N
Thermosphaeroma subequalum	Th_spp_056	Lower Madison Falls, Brewster County, TX, USA	-	-	Unknown	Unknown	0.58 ng/μL	Y
Thermosphaeroma subequalum	Th_spp_058	Panther Rapids, Brewster County, TX, USA	-	-	Unknown	Unknown	1.01 ng/μL	Y
Cassidinea ovalis	AGC-709.1	DeLeon Springs, Volusia County, FL, USA	29.13492	-81.36327	Andrew G. Cannizzaro	9/22/2017	5.28 ng/μL	Y
Hyalella cretae	AGC-379.2	Chalk Spring, Nye County, NV, USA	36.44904	-116.31506	Corey Lange	11/24/2020	3.06 ng/μL	Y
Hyalella cretae	AGC-388.3	Point of Rocks Spring #42, Nye County, NV, USA	36.40258	-116.27348	Corey Lange	12/11/2020	8.90 ng/μL	Y
Hyalella muerta	AGC-436.2	West Travertine Springs, Inyo County, CA, USA	36.444315	-116.83004	Corey Lange	4/10/2021	1.13 ng/μL	Y
Hyalella sandra	AGC-437.1	East Travertine Springs, Inyo County, CA, USA	36.44262	-116.827082	Corey Lange	4/10/2021	2.92 ng/μL	Y

Hyalella wakulla	AGC-986.1	Wakulla River, Wakulla County, FL, USA	30.23527	-84.3014	T.R. Sawicki, R.A. Long	3/7/2020	8.98 ng/µL	N
Stygobromus tenuis	AGC-592.1	Caledon State Park, Site 4, King George County, VA, USA	-	-	C. Hobson	4/14/2017	8.08 ng/µL	Y
Bactrurus mucronatus	AGC-192.2	Well in Enon, Clark County, OH, USA	39.881194	-83.915994	Mike Kammer	9/2/2021	19.4 ng/µL	N
Crangonyx bousfieldi	AGC-601.1	French Park, Hamilton County, OH, USA	39.20096	-84.41782	Andrew G. Cannizzaro	5/7/2022	53.8 ng/µL	N
Crangonyx cf. *floridanus*	AGC-524.3	Lee Highway Seep, Jackson County, AL, USA	34.61894	-86.14123	Andrew G. Cannizzaro	3/25/2022	58.4 ng/µL	N
Gammarus balmorhea	AGC-181.2	San Solomon Spring, Reeves County, TX, USA	30.94436	-103.78597	Ashley Walters	6/1/2013	32.6 ng/µL	N
Gammarus lacustris	AGC 354.3	Cane Spring, Nye County, NV, USA	38.22572	-116.18523	Corey Lange	10/11/2020	20.6 ng/µL	N

MOLECULAR ECOLOGY RESOURCES

Table S2. Additional peracarid taxa included in the *in-vitro* analysis obtained from the NCBI Sequence Read Archive, along with unique UCE loci recovered under the +10 *P. hawaiensis* probe set.

Order	Family	Species	Accession #	Loci Recovered
Cumacea	Diastylidae	*Diastylis cornuta*	R25406671	1,048
Tanaidacea	Pseudotanaidae	*Pseudotanais* sp.	ERX14599716	1,421
Isopoda	Munnopsidae	*Notopais cryophila*	SRX13146481	2,321
Isopoda	Asellidae	*Asellus aquaticus*	SRX10785126	2,974
Isopoda	Cymothidae	*Ceratothoa italica*	SRR11560933	1,737
Amphipoda	Phronimidae	*Phronima sedentaria*	SRX21882092	819
Amphipoda	Cyamidae	*Cyamus boopis*	SRX12200346	1,607
Amphipoda	Cyphocarididae	*Cyphocaris challengeri*	SRX18928790	1,157
Amphipoda	Eusiridae	*Eusirus giganteus*	SRX13936486	1,093
Amphipoda	Caprellidae	*Caprella scaura*	DRX682979	1,449
